# Comparative genomic and transcriptional analyses of the carbohydrate-active enzymes and secretomes of phytopathogenic fungi reveal their significant roles during infection and development

**DOI:** 10.1038/srep15565

**Published:** 2015-11-04

**Authors:** Xueliang Lyu, Cuicui Shen, Yanping Fu, Jiatao Xie, Daohong Jiang, Guoqing Li, Jiasen Cheng

**Affiliations:** 1 State Key Laboratory of Agricultural Microbiology, Huazhong Agricultural University, Wuhan 430070, Hubei Province, China; 2 The Provincial Key Lab of Plant Pathology of Hubei Province, College of Plant Science and Technology, Huazhong Agricultural University, Wuhan 430070, Hubei Province, China

## Abstract

Our comparative genomic analysis showed that the numbers of plant cell wall (PCW)- and fungal cell wall (FCW)-degradation-associated carbohydrate-active enzymes (CAZymes) in necrotrophic and hemibiotrophic fungi are significantly larger than that in most biotrophic fungi. However, our transcriptional analyses of CAZyme-encoding genes in *Melampsora larici-populina*, *Puccinia graminis* and *Sclerotinia sclerotiorum* showed that many genes encoding PCW- and FCW-degradation-associated CAZymes were significantly up-regulated during the infection of both necrotrophic fungi and biotrophic fungi, indicating an existence of a universal mechanism underlying PCW degradation and FCW reorganization or modification, which are both intimately involved in necrotrophic and biotrophic fungal infection. Furthermore, our results showed that the FCW reorganization or modification was also related to the fungal development. Additionally, our transcriptional analysis of the secretome of *S. sclerotiorum* showed that many secreted protein-encoding genes were dramatically induced during infection. Among them, a small, cysteine-rich protein SsCVNH was experimentally confirmed to be essential for the virulence and sclerotial development, indicating that the small secreted proteins might also play crucial roles as potential effectors in host-non-specific necrotrophic fungi.

Many fungal carbohydrate-active enzymes (CAZymes) and secreted proteins directly participate in the interactions between phytopathogenic fungi and their hosts. Among the secretome, CAZymes are a large group, but not all the members of this group are secreted proteins. CAZymes are responsible for the breakdown, biosynthesis or modification of glycoconjugates and oligo- and polysaccharides by degrading, modifying, or creating glycosidic bonds. They participate in many important biological processes, including cell wall synthesis, signaling, and energy production^1^. Currently, there are four main classes of CAZymes: glycosyltransferases (GTs), glycoside hydrolases (GHs), polysaccharide lyases (PLs) and carbohydrate esterases (CEs)^2^. The latter three classes of CAZymes are often known as cell wall degrading enzymes (CWDEs) because they play central roles in plant cell wall (PCW) decomposition by fungi and bacteria^3^. There are many proteins with carbohydrate-binding modules (CBMs) that can potentiate CAZyme activities by targeting and promoting a prolonged interaction with the substrate, although they have no catalytic activity *per se* except some CAZymes with CBMs^2^. Generally speaking, PCW consists of carbohydrate (including cellulose, hemicellulose, pectin and lignin), structural proteins, and aromatic compounds^4^,^5^, while fungal cell wall (FCW) is primarily composed of chitin, glucans, mannans and glycoproteins^6^. Owing to the distinct components of PCW and FCW, fungi have developed two categories of CAZymes: PCW- and FCW-degrading CAZymes, which have different enzyme activities and substrates, to degrade or modify PCW and FCW respectively. Because breakthrough of PCW is an essential step for the colonization of host tissues for all phytopathogens, CWDEs, such as pectinases and xylanases, have been identified as important pathogenicity or virulence factors^7^,^8^. A large number of CAZyme-encoding genes in the genomes of phytopathogenic fungi are associated with PCW degradation. It is well known that PCW-degrading CAZymes play crucial roles during fungal infection. There are a large number of genes encoding FCW-degrading enzymes in phytopathogenic fungal genomes, however, the expression patterns and the exact roles of these FCW-degrading CAZymes during fungal infection and development are unknown.

*Sclerotinia sclerotiorum* (Lib.) de Bary is a necrotrophic, phytopathogenic fungus with a remarkably broad host range and worldwide distribution. At least 408 described species of plants from 278 genera in 75 families, including many economically important crops such as rapeseed (*Brassica* spp.), sunflowers, soybeans and peanuts, are susceptible to this pathogen^9^. White mold caused by this pathogen often leads to a momentous loss of crop production. Importantly, for many important crops such as rapeseed, there are almost no resistant cultivars that can be used for production. Thus, *Sclerotinia* disease is difficult to control and has raised considerable concern. There are several key steps in the life cycle of *S. sclerotiorum*, including vegetative growth, infection, sclerotial development, sclerotial myceliogenic germination, sclerotial carpogenic germination and apothecium formation (stipe). These steps are all important for the lifecycle of *S. sclerotiorum*. For example, the sclerotium produced by *S. sclerotiorum* is a pigmented, hard, asexual resting structure that is capable of surviving for years in soil^10^,^11^,^12^. Sclerotia germinate myceliogenically to produce saprophytic or infectious mycelia or carpogenically to form apothecia. Airborne ascospores derived from apothecia are the principal source of primary inocula and the most important means of *S. sclerotiorum* dispersal^13^,^14^,^15^.

The secretomes play crucial roles during plant-pathogen interactions. Notably, most effectors of fungi and oomycetes are small secreted proteins (especially small, secreted, cysteine-rich proteins)^16^,^17^,^18^,^19^,^20^, with the exception of some non-proteinaceous toxins and secondary metabolites. Many effectors have been well documented in host-specific necrotrophic fungi, hemibiotrophic fungi, biotrophic fungi and oomycetes^16^,^17^,^18^,^19^,^20^. However, most of the secreted proteins in host-non-specific, typically necrotrophic fungi with a remarkably broad host range, such as *S. sclerotiorum*, are poorly understood. Secretome analysis revealed large numbers of predicted effector candidates in the genome of *S. sclerotiorum*^21^, and recent studies demonstrated that the non-CWDE secreted proteins played crucial roles during infection^22^,^23^. However, the large-scale transcriptional responses of genes encoding the secretome during the infection, vegetative and reproductive developmental stages of *S. sclerotiorum* and the exact biological functions of most of these effector candidates are unknown.

In this study, we compared the numbers of the CAZyme-encoding genes in the genomes of different phytopathogenic fungi and investigated the global transcriptional responses of the CAZyme-encoding genes and secretome-encoding genes during the infection and development of phytopathogenic fungi. To confirm our hypotheses, 16 genomes of phytopathogenic fungi (including biotrophs, hemibiotrophs and necrotrophs) and three expression profiles [including two reported microarray data of rust fungi and one digital gene expression profiling (DGE) data of *S. sclerotiorum*] were chosen for comparative analysis. Additionally, as an example, one small, cysteine-rich, secreted protein (SsCVNH) was chosen for functional studies. Our results increase the understanding of the roles of CAZymes and the secretomes in the infection and development of phytopathogenic fungi and facilitate the discovery of novel effector candidates in host-non-specific necrotrophic fungi.

## Results

### Comparative genomic analysis of the CAZymes in phytopathogenic fungi

**Figure 1:**
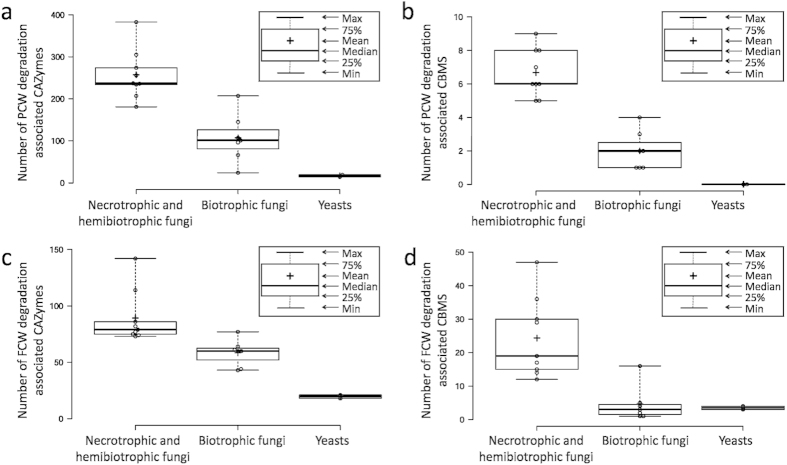
Comparative analysis of CAZymes and related CBMs in necrotrophic and hemibiotrophic fungi, biotrophic fungi and two yeasts. The numbers of PCW- (**a**) and FCW- (**c**) degradation-associated CAZymes and respective related CBMs ((**b**,**d**) respectively) were plotted for necrotrophic and hemibiotrophic fungi, biotrophic fungi and two yeasts. The numbers of PCW- and FCW-degradation-associated CAZymes and related CBMs showed significant differences among necrotrophic and hemibiotrophic fungi, biotrophic fungi and two yeasts. Differentiation was supported by the t-test at a significance level of P < 0.05.

Considering the representativeness and accessibility of available genome data, nine genomes of hemibiotrophic and necrotrophic fungi (*Magnaporthe oryzae*, *Fusarium graminearum*, *F. oxysporum*, *F. verticillioides*, *Phaeosphaeria nodorum*, *Pyrenophora tritici-repentis*, *Verticillium dahliae*, *Botrytis cinerea* and *S. sclerotiorum*), seven genomes of biotrophic fungi (*Blumeria graminis*, *Ustilago maydis*, *Cladosporium fulvum*, *Melampsora larici-populina*, *Puccinia graminis*, *P. triticina* and *P. striiformis*) and two yeast genomes (*Saccharomyces cerevisiae* and *Schizosaccharomyces pombe*) were chosen for the comparative analysis of CAZymes. Our results showed that the numbers of genes encoding PCW- and FCW-degradation-associated CAZymes and their respective related CBMs in necrotrophic and hemibiotrophic fungi were both significantly larger than that in most of biotrophic fungi except for *C. fulvum* (Fig. 1, Table S1), indicating both PCW- and FCW-degradation-associated CAZymes may play crucial roles during the infection and (or) development of necrotrophic and hemibiotrophic fungi.

### DGE of S. sclerotiorum

To explore the expression patterns of CAZyme-encoding genes during the infection and development of necrotrophic fungi, we used DGE based on deep sequencing technology to illuminate the transcriptional responses of CAZyme-encoding genes during the infection stage and different developmental stages (including vegetative growth, sclerotial development, sclerotial myceliogenic germination, sclerotial carpogenic germination and apothecium formation) of the necrotrophic fungus *S. sclerotiorum*, which is used as an example. Sequencing quality evaluation and sequencing saturation analyses guaranteed the data quality for further DGE analysis (Figure S1 and S2). Differentially expressed genes were identified during the various developmental stages compared with the vegetative growth stage (Figure S3). To validate the DGE data, twelve genes were selected randomly for quantitative reverse transcription PCR (qRT-PCR) analysis. The data were presented as fold changes in gene expression normalized to the β-tubulin gene^24^,^25^,^26^. Pearson’s correlation coefficient (R value) was used to measure the consistency of the qRT-PCR and DGE data. The R values ranged from 0.88 to 1.00, indicating the qRT-PCR results were strongly correlated with the DGE data (Figure S4). These results showed that the expression patterns of the twelve genes in the DGE and qRT-PCR data were accordant and our DGE data was reliable for further transcriptional analysis.

### The expression patterns of CAZyme-encoding genes in *S. sclerotiorum*

**Figure 2:**
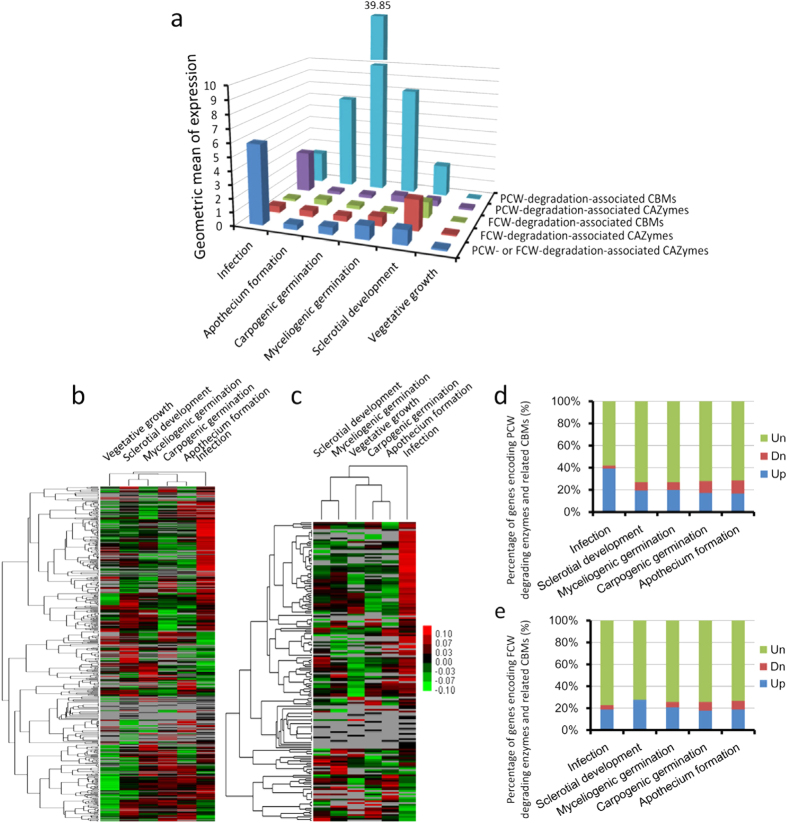
Transcriptional analysis of genes encoding CAZymes and related CBMs during different developmental stages of *S. sclerotiorum*. (**a**) The expression levels of genes encoding PCW- and FCW-degradation-associated CAZymes and respective related CBMs during different developmental stages. The expression levels were measured using the geometric mean of the TPM values of all genes in corresponding groups. To calculate the geometric mean of TPM values, all the TPM values of 0 were replaced by 0.001. (**b**) Gene expression cluster analysis of genes encoding PCW-degradation-associated CAZymes and related CBMs. (**c**) Gene expression cluster analysis of genes encoding FCW-degradation-associated CAZymes and related CBMs. The TPM values were used for the gene expression cluster analysis. Red, green and grey indicate high expression, low expression and no expression, respectively; For each heat map: top, stage tree; left, gene tree. Expression values are indicated in log2 scale. Column diagrams show the proportions of differentially expressed genes and unchanged expressed genes encoding PCW- (**d**) and FCW- (**e**) degradation-associated CAZymes and respective related CBMs, during different developmental stages, compared with the vegetative growth stage of *S. sclerotiorum*. The differentially expressed genes in the DGE data were identified with the threshold of |log_2_Ratio| ≥ 1 and FDR ≤ 0.001. Up, Down and Un indicate the up-regulated, down-regulated and unchanged expressed genes, respectively.

**Figure 3:**
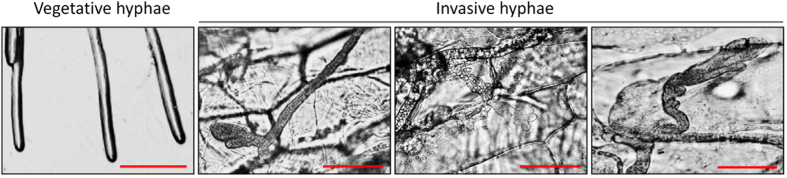
Morphology of the vegetative and invasive hyphal tips of *S. sclerotiorum*. Cellophane on PDA plates and onion bulb epidermis were inoculated with the *S. sclerotiorum* wild-type strain for 36 h at 20 °C prior to microscopic examination. Microscopic observation showed that the invasive hyphal tips were thicker than the vegetative hyphal tips and that their morphology was blistered, swollen and malformed. Scale bar = 100 μm.

Among the 501 identified CAZyme-encoding genes in *S. sclerotiorum*, the expression of 315 was significantly regulated during different developmental stages, compared with vegetative growth stage (Table S2a). Our result showed that the geometric mean of the expression values of the PCW- and FCW-degrading CAZyme-encoding genes and their respective related CBMs showed dramatic induction during the infection and various developmental stages of *S. sclerotiorum* (Fig. 2a), indicating both of the PCW- and FCW-degrading CAZymes were activated during these biological processes. As expected, gene expression cluster analysis showed that a large number of genes encoding PCW-degradation-associated CAZymes and related CBMs were significantly up-regulated during infection (Fig. 2b). For example, 73 (39.2%) out of a total of 186 genes encoding PCW-degradation-associated CAZymes and related CBMs, were significantly up-regulated during infection (Fig. 2d, Table S3). This result confirmed the important roles of plant CWDEs during infection. Additionally, a large number of genes encoding FCW-degradation-associated CAZymes and related CBMs were also dramatically induced during infection (Fig. 2c). For example, 19 (18.8%) from a total of 101 genes encoding FCW-degradation-associated CAZymes and related CBMs, were significantly up-regulated during infection (Fig. 2e, Table S3). Microscopic observation showed that the invasive hyphal tips were clearly different from the vegetative hyphal tips: the latter were thinner and their morphology was normal, whereas the former were thicker and their morphology was blistered, swollen and malformed (Fig. 3). This result further suggested FCW reorganization or modification was intimately involved in the infection processes of necrotrophic fungi. During the vegetative and reproductive developmental stages, there are 36 (19.4%), 37 (19.9%), 32 (17.2%) and 31 (16.7%) out of a total of 186 genes encoding PCW-degrading enzymes and related CBMs which were significantly up-regulated during sclerotial development, sclerotial myceliogenic germination, sclerotial carpogenic germination and apothecium formation, respectively, compared with the vegetative growth stage (Fig. 2d, Table S3), indicating the expression of these genes encoding PCW-degrading enzymes and related CBMs was induced not by specific plant factors but by the developmental regulation of *S. sclerotiorum* or by tough environmental factors which are unfavorable for vegetative growth (such as nutrient starvation). Notably, a large number of genes encoding FCW-degrading CAZymes and related CBMs were also significantly induced during the vegetative and reproductive developmental stages of *S. sclerotiorum*. For example, there are 28 (27.7%), 21 (20.8%), 18 (17.8%) and 19 (18.8%) from a total of 101 genes encoding FCW-degrading enzymes and related CBMs that were significantly up-regulated during sclerotial development, sclerotial myceliogenic germination, sclerotial carpogenic germination and apothecium formation, respectively, compared with the vegetative growth stage (Fig. 2e, Table S3). These results indicated that the FCW reorganization or rearrangement also played important roles in the vegetative and reproductive development of *S. sclerotiorum*, and the FCW was in a state of dynamic change during various developmental stages. Taken together, the CAZyme-encoding genes exhibited diverse expression patterns during the infection and multiple developmental stages of *S. sclerotiorum*, compared with the vegetative stage, indicating that the CAZymes might play versatile roles in these developmental processes.

### Transcriptional analyses of CAZyme-encoding genes in *M. larici-populina* and *P. graminis*

**Figure 4:**
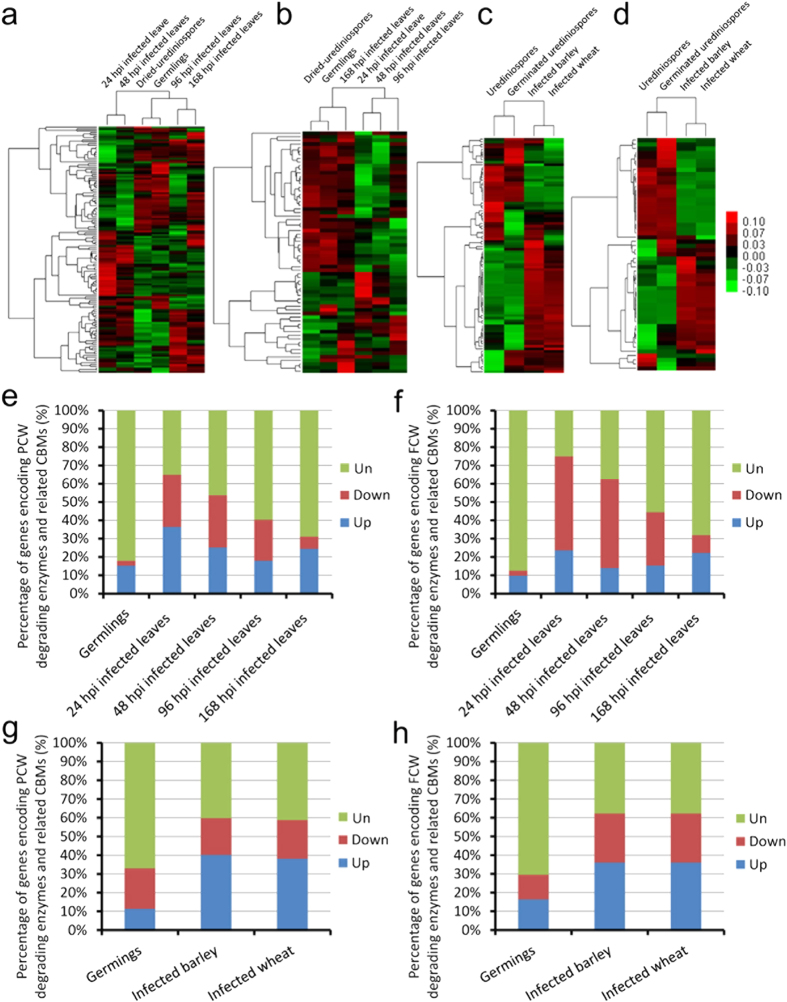
Transcriptional analysis of genes encoding CAZymes and related CBMs in *M. larici-populina* and *P. graminis* during urediniospore germination and infection. Expression cluster analysis of genes encoding PCW- (**a**) and FCW- (**b**) degradation-associated CAZymes and respective related CBMs in *M. larici-populina* and expression cluster analysis of genes encoding PCW- (**c**) and FCW- (**d**) degradation-associated CAZymes and respective related CBMs in *P. graminis* were performed based on the expression average values of three independently repeated tests^27^,^28^. Red and green indicate high expression and low expression respectively. For each heat map: top, stage tree; left, gene tree. Expression values are indicated in log2 scale. Column diagrams show the proportions of differentially expressed genes encoding PCW- (**e**) and FCW- (**f**) degradation-associated CAZymes and respective related CBMs, during *M. larici-populina* urediniospore germination and different infection stages on poplar leaves, compared with dried-urediniospores *in vitro*. Column diagrams show the proportions of differentially expressed genes encoding PCW- (**g**) and FCW- (**h**) degradation-associated CAZymes and respective related CBMs during *P. graminis* urediniospore germination and infection on wheat and barley, respectively, compared with urediniospores *in vitro*. The differentially expressed genes in the microarray data were identified with the threshold of |Fold change |  2 and an adjusted p-value < 0.05. Up, Down and Un indicate the up-regulated, down-regulated and unchanged expressed genes, respectively.

To detect the expression patterns of CAZyme-encoding genes during the infection of phytopathogenic biotrophic fungi, we examined the documented gene expression profiles (microarray data) during the infection of *M. larici-populina*^27^ and *P. graminis*^28^ urediniospores, which are used as examples. The results showed that the expression of a large number of genes encoding CAZymes and related CBMs in *M. larici-populina* and *P. graminis* was significantly regulated during infection. In details, 75 (49.7%) out of a total of 151 genes encoding PCW-degradation-associated CAZymes and related CBMs were significantly induced during at least one infection stage of *M. larici-populina* urediniospores on poplar leaves, compared with the stage of dried-urediniospores *in vitro* (Table S4a), the proportion varied from 17.9% to 36.4% during different infection stages (Fig. 4e, Table S4a). There are 39 (40.2%) and 37 (38.1%) out of a total of 97 genes encoding PCW-degradation-associated CAZymes and related CBMs that were significantly induced during the infection of *P. graminis* urediniospores on wheat and barley, respectively, compared with the stage of urediniospores *in vitro* (Fig. 4g, Table S4b). Surprisingly, many genes encoding FCW-degrading CAZymes and related CBMs in *M. larici-populina* and *P. graminis* were also significantly regulated during infection. In details, there are 26 (36.1%) from a total of 72 genes encoding FCW-degradation-associated CAZymes and related CBMs which were significantly induced during at least one infection stage of *M. larici-populina* urediniospores on poplar leaves, compared with the stage of dried-urediniospores *in vitro* (Table S4a), the proportion varied from 13.9% to 23.6% during different infection stages (Fig. 4f). There are 22 (36.1%) and 22 (36.1%) from a total of 61 genes encoding FCW-degradation-associated CAZymes and related CBMs that were significantly induced during the infection of *P. graminis* urediniospores on wheat and barley, respectively, compared with the stage of urediniospores *in vitro* (Fig. 4h, Table S4b). These results indicated that the PCW degradation and FCW reorganization or modification might also be intimately involved in the infection processes of biotrophic fungi, although the numbers of genes encoding PCW and FCW-degradation associated CAZymes in biotrophic fungi were smaller than those in necrotrophic fungi.

### Transcriptional analysis of the secretome of *S. sclerotiorum*

We compared the total numbers of secreted proteins, small secreted proteins and cysteine-rich, small secreted proteins from the 18 fungi described above, respectively. The result showed *P. striiformis*, *M. larici-populina*, *F. verticillioides*, *M. oryzae*, *P. graminis*, *F. oxysporum* and *P. triticina* had more secreted proteins, especially small secreted proteins, compared with the other species (Figure S5). To decipher the biological functions of the *S. sclerotiorum* secretome, the gene expression patterns of the secretome were clustered based on our DGE data. The result showed that many genes encoding secreted proteins were significantly regulated during the infection, sclerotial development, sclerotial myceliogenic germination, sclerotial carpogenic germination and apothecium formation stages, compared with the vegetative growth stage (Figure S6, Table S2b), indicating their diversified roles during these biological processes. Gene functional enrichment analysis was performed on the significantly up-regulated genes encoding secreted proteins during different developmental stages. Many Funcat functional categories and gene ontology (GO) items were enriched during various developmental stages, especially during the sclerotial development and infection stages (Figure S7, Table S5), indicating their important and diverse roles in these biological processes. Interestingly, compared with the vegetative growth stage, the “cell wall” associated functional modules (such as “cell wall organization”, “fungal-type cell wall” and “cell wall modification”) were significantly over-represented during different developmental stages (Figure S7, Table S5), which is in accord with our hypothesis that the FCW is in a state of dynamic change during various developmental stages. Additionally, the results showed the PCW- and FCW-degradation associated functional modules (such as “extracellular polysaccharide degradation”, “extracellular lignin degradation”, “cellulose binding”, “chitin binding”, “hydrolase activity”, “cellulase activity”, “cutinase activity”, “polygalacturonase activity”, “beta-galactosidase activity” and “mannan endo-1,6-alpha-mannosidase activity”) were also over-represented during different developmental stages (Figure S7, Table S5), intimating the significant roles of CAZymes in corresponding biological processes.

### SsCVNH is a predicted cysteine-rich, small secreted protein with a CVNH domain

**Figure 5:**
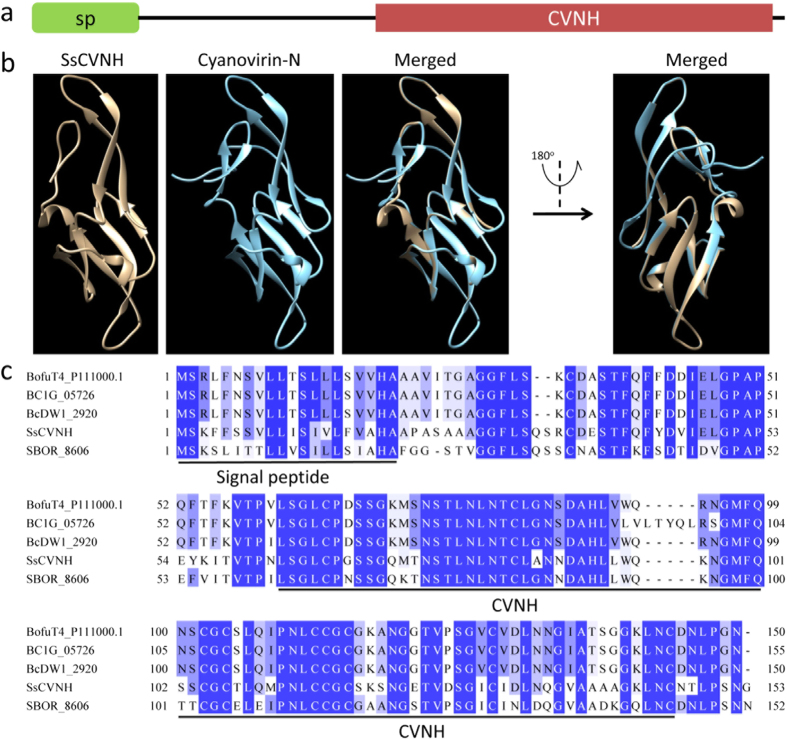
SsCVNH has a conserved CVNH domain. (**a**) Domain architecture of SsCVNH. Protein sequences are indicated by thick lines. Signal peptide (sp) and CVNH domain are indicated by boxes. (**b**) Representation of the three-dimensional structure of cyanovirin-N^33^ and predicted SsCVNH. A total of 85 residues (56% of the SsCVNH) were modelled with 98.8% confidence by the single highest scoring template cyanovirin-N (d1n02a) in the Phyre database. (**c**) Multiple sequence alignment of amino acid sequences of CVNHs from *Sclerotinia* and *Botryotinia*. Protein sequences from top to bottom are derived from *B. cinerea* T4, *B. cinerea* B05.10, *B. cinerea* BcDW1, *S. sclerotiorum* Ep-1PNA367 and *Sclerotinia borealis* F-4157, respectively.

To experimentally validate the important roles of small secreted proteins during the infection, vegetative and reproductive developmental stages of *S. sclerotiorum*, a predicted secreted protein-encoding gene (*SS1G_02904*) was selected as an example for further study. We chose this gene because of the following reasons: (i) In planta expression analysis can help to unearth candidate effector genes^18^ and *SS1G_02904* had the highest expression fold change during the infection in our DGE data; (ii) Many fungal effectors are cysteine-rich, small secreted proteins^17^,^29^, and SS1G_02904 consists of 153 aa and contains 10 cysteine residues that account for more than 6.5% of the total amino acids; (iii) Some fungal effectors show conserved protein domains, and SS1G_02904 was identified as an effector candidate that shares 35% identity with a *Colletotrichum hingginsianum* effector candidate^21^,^30^. SS1G_02904 is a predicted secreted protein with a signal peptide (1–20 aa) in its N-terminus and a CVNH domain (pfam08881, E-value = 2.65e-06) in its C-terminus (Fig. 5a); hence, it is designated as SsCVNH. The CVNH domain corresponds to a carbohydrate-binding module^31^,^32^. The predicted three-dimensional structure of SsCVNH is highly similar to the structure of cyanovirin-N^33^ (Fig. 5b). Although the CVNH domain was found to be widely distributed in cyanobacteria, filamentous ascomycetes and the fern *Ceratopteris richardii*^34^, protein sequence similarity search (blastp) result showed that SsCVNH had homologs only in *Sclerotinia* and *Botryotinia*, using an E-value lower than 1e-6 as the threshold (Fig. 5c). These results indicated that the three-dimensional structure of the CVNH domain was more evolutionarily conserved than the protein sequence.

### The expression patterns of *SsCVNH*

**Figure 6:**
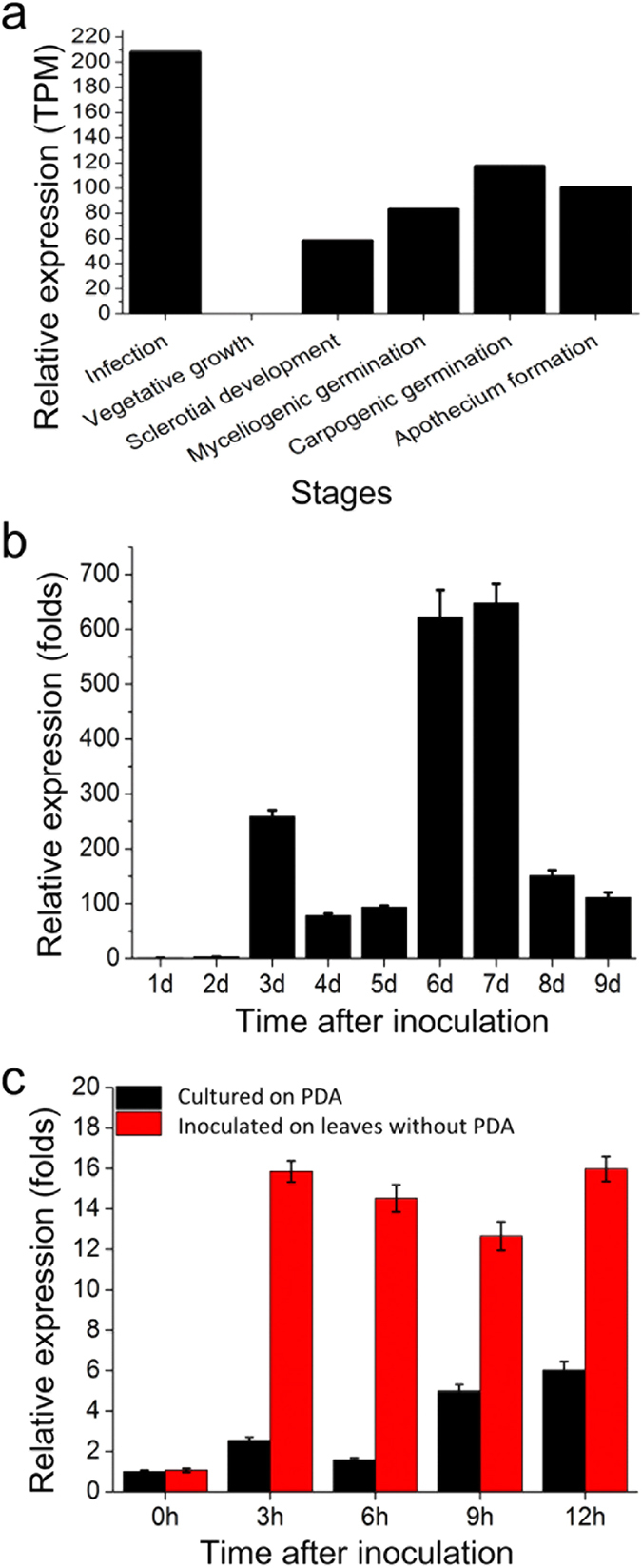
The expression patterns of *SsCVNH* in the wild-type strain. (**a**) The relative expression of *SsCVNH* during different developmental stages in the DGE data. (**b**) The relative expression of *SsCVNH* on PDA at 20 °C for 1–9 days. The expression level on the first day was set at 1.0. (**c**) The relative expression of *SsCVNH* post inoculation of the wild-type strain on *A. thaliana* leaves (red columns) and on PDA (dark columns) for 0–12 h. The expression level at 0 hpi on PDA was set at 1.0. The expression level of β-tubulin was used to normalize *SsCVNH* expression. The values are presented as the means ± s.d.

DGE data showed that *SsCVNH* was not expressed during the vegetative growth stage and was significantly induced during the stages of sclerotial development, sclerotial myceliogenic germination, sclerotial carpogenic germination, apothecium formation and infection (Fig. 6a). QRT-PCR analysis further showed that the expression of *SsCVNH* was significantly up-regulated during the initial stage of sclerotial development at 3 days post-inoculation (dpi) and peaked during the mature stage of sclerotial development at 7 dpi. The expression change of this gene reached more than 600-fold during sclerotial development (Fig. 6b). When pure, actively growing hyphal fragments of *S. sclerotiorum* without any culture medium were inoculated onto *Arabidopsis thaliana* (Col-0) leaves, the transcripts of *SsCVNH* rapidly increased by more than 15-fold during the early infection stage at 3 hours post-inoculation (hpi) and maintained at high expression levels during the later infection stages (Fig. 6c). These results were coincident with the DGE data, suggesting that *SsCVNH* may be involved in the infection, vegetative and reproductive development of *S. sclerotiorum*.

### SsCVNH is secreted from the hyphal tips

**Figure 7:**
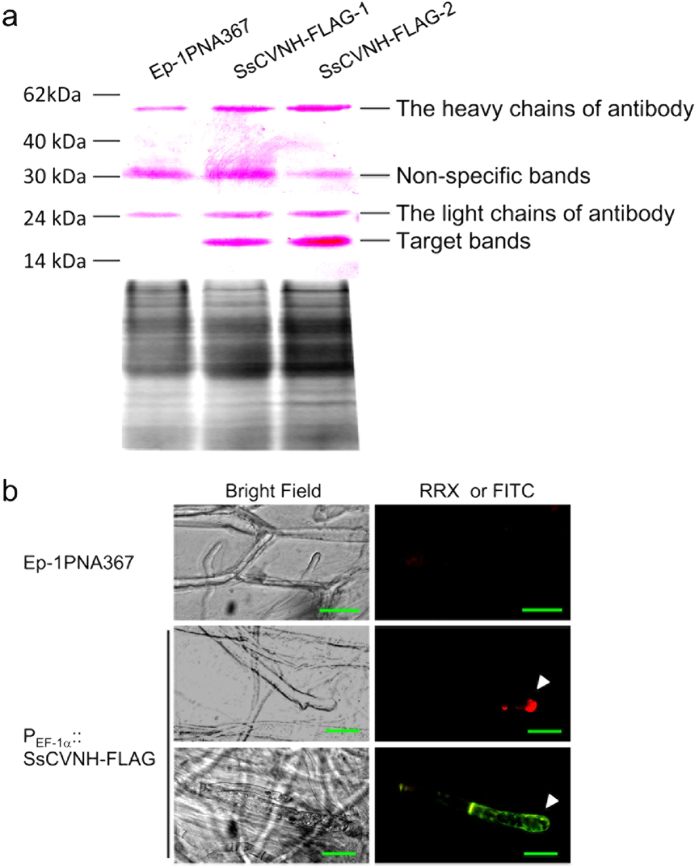
SsCVNH was secreted from the hyphal tips during infection. (**a**) Western blot analysis showed that SsCVNH-FLAG could be detected in the engineered strains. SDS-polyacrylamide gel electrophoresis showed the equal loading amounts of total proteins used for the western blot analysis. Alkaline phosphatase-conjugated secondary antibody detected an approximately 17 kDa band in the SsCVNH-FLAG-engineered strains but not in the wild-type strain. (**b**) Immunofluorescence detection of SsCVNH-FLAG during infection. Onion bulb epidermis was inoculated with SsCVNH-FLAG-engineered strains for 36 h at 20 °C. Two different secondary antibodies conjugated with RRX or FITC respectively, were used independently to exhibit the specificity of fluorescence signal. The fluorescence of both RRX (red) and FITC (green) was detected in the SsCVNH-FLAG-engineered strains, but not in the wild-type strain. Arrows show the concentrated distribution of SsCVNH in the hyphal tips during infection. Scale bar = 50 μm.

Bioinformatics analysis revealed that SsCVNH might be a secreted protein due to the presence of a signal peptide in its N-terminus. An immunofluorescence technique was used to confirm this hypothesis. FLAG-tagged SsCVNH strains under the control of the P_EF_ − 1α promoter were engineered. Western blot analysis showed FLAG-tagged SsCVNH could be detected in the hyphae of these engineered strains (Fig. 7a). The mycelia of the engineered SsCVNH-FLAG strains were inoculated on the onion bulb lower epidermis prior to live cell imaging of the infected onion epidermal cells to examine the subcellular localization of SsCVNH during infection. To eliminate the possible interference of the autofluorescence of plant or fungal tissues, two different secondary antibodies conjugated with rhodamine red-X (RRX) or fluorescein isothiocyanate (FITC) were used independently to exhibit the specificity of fluorescence signal. The result showed fluorescence could be observed in the hyphal tips of the SsCVNH-FLAG engineered strains. No fluorescence was observed in the wild-type strain. Similar results were obtained with different antibodies tagged with FITC or RRX (Fig. 7b), indicating that SsCVNH was indeed a secreted protein that was secreted from the hyphal tips during the infection of *S. sclerotiorum*.

### *SsCVNH*-silenced transformants showed significantly reduced virulence and abnormal sclerotial development

**Figure 8:**
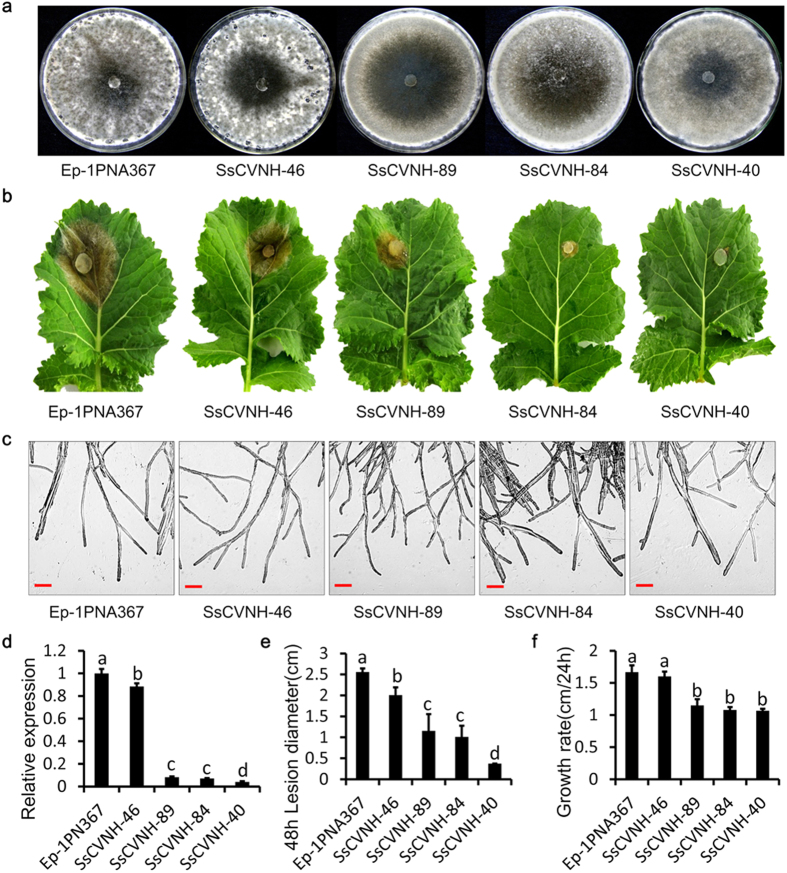
The phenotypes of *SsCVNH*-silenced transformants. (**a**) Colony morphology in *SsCVNH*-silenced transformants. Colonies were grown on PDA for 10 days at 20 °C. (**b**) *SsCVNH*-silenced transformants showing significantly reduced virulence on detached rapeseed (*Brassica napus*) leaves. Virulence was evaluated based on the lesion diameter at 20 °C for 48 h. (**c**) Comparison of the hyphal tips of *SsCVNH*-silenced transformants and the wild-type strain. Scale bar = 100 μm. (**d**) Expression of *SsCVNH* in silenced transformants was determined by qRT-PCR. The expression levels of β-tubulin were used to normalize *SsCVNH* expression. The expression level of *SsCVNH* in the wild-type strain was set at 1.0. (**e**) Comparison of the lesion diameters of the silenced transformants and the wild-type strain. (**f**) Comparison of the growth rates of the silenced transformants and the wild-type strain. In all experiments, three independent replications were performed. The values are presented as the means ± s.d. Different letters in the graph indicate significant differences while same letters indicate no significant differences, P = 0.05.

Due to the presence of the multi-nucleated cells in *S. sclerotiorum*, RNAi technology was used to characterize the biological functions of *SsCVNH*. QRT-PCR was used to examine the transcript accumulation in *SsCVNH*-silenced transformants. The results showed that the sclerotial development of the transformants (SsCVNH-89, SsCVNH-84 and SsCVNH-40) with dramatically reduced *SsCVNH* expression was completely blocked on PDA medium at 20 °C. However, the wild-type strain and the transformant SsCVNH-46 with slightly reduced *SsCVNH* expression showed normal sclerotial development (Fig. 8a,d). The growth rate of the silenced transformants was also significantly reduced, although there was no obvious difference between the morphology of the hyphal tips of the silenced transformants and the wild-type strain (Fig. 8c,f). The virulence of the *SsCVNH*-silenced transformants was significantly reduced, and only small lesions developed on the detached leaves of *Brassica napus* at 2 dpi. Furthermore, the decrease in virulence was positively correlated with the silencing efficiency of different transformants (Fig. 8b,e). Similar result was observed when the *SsCVNH*-silenced transformants were inoculated on detached tomato leaves (Figure S8), indicating that the virulence reduction was not host species-specific. These results indicated that *SsCVNH* played crucial roles in the virulence, sclerotial development and growth rate of *S. sclerotiorum*. In conclusion, our results indicated that small secreted proteins in typically necrotrophic fungi might function as potential effectors similar to those in hemibiotrophs and biotrophs.

## Discussion

To date, whether the phytopathogenic fungi use FCW-degrading enzymes for the degradation or modification of their own cell walls, the walls of antagonistic fungi, or for plant polysaccharides during infection is unknown^35^. Our results indicated that the expression levels of many genes encoding FCW-degrading enzymes were significantly regulated during the infection of phytopathogenic fungi. Meanwhile, our results indicated different kinds of FCW-degrading enzymes participated in this biological process and their proportions varied during different infection stages, indicating the FCW reorganization or modification process is sophisticatedly and stringently regulated. The present results support the hypothesis that these FCW-degrading enzymes may play significant roles in shaping their own cell walls because the FCW reorganization or remodeling could be clearly observed during infection. The FCW reorganization or rearrangement itself may be essential for phytopathogenic fungal infection. This hypothesis may also be adapted to the development of phytopathogenic fungi because the FCW components are obviously different in various tissues during different developmental stages. Our results indicated that the FCW was in a state of dynamic change during the infection and development of phytopathogenic fungi, which is consistent with the previous studies on FCW dynamics^6^,^36^. Additionally, our results indicated that the PCW degradation and FCW reorganization are intimately involved in both necrotrophic and biotrophic fungal infection. But the total numbers of genes encoding PCW- and FCW-degrading CAZymes and associated CBMs in necrotrophic and hemibiotrophic fungi are significantly larger than that in most biotrophic fungi. Furthermore, the proportions of differentially expressed CAZyme-encoding genes are also obviously different during infection, indicating the capacity of PCW degradation and FCW reorganization is different in necrotrophic, hemibiotrophic and biotrophic fungi. This is consistent with the lifestyles of different types of fungi, because the nutrient acquisition of necrotrophic and hemibiotrophic fungi is based on host cell killing at the last stage of infection^37^, while biotrophic fungi absorb nutrients from living cells^38^.

Our result showed a total of 235 secreted protein-encoding genes were significantly up-regulated during infection (Table S2b). These strongly induced genes are likely to represent “key offensive or repressive forces” or “effector candidates” in *S. sclerotiorum* that target the plant defense system during the early stages of infection. Notably, many proteinaceous fungal effectors are cysteine-rich, small secreted proteins^16^,^17^,^29^. In this study, SsCVNH was used as an example to elucidate the significant roles of these types of effector candidates during the infection, vegetative and reproductive development of *S. sclerotiorum*. In many reports, most effectors or small secreted proteins seemed to have little effect on pathogen development. Interestingly, this may not be the case in *S. sclerotiorum* because, to date, at least two small secreted proteins (SSITL and Ss-Caf1) have been reported to play important roles in the sclerotial development of *S. sclerotiorum*^22^,^23^. Similarly, our results demonstrated that SsCVNH also played significant roles in sclerotial development, not only in infection. Additionally, in our large-scale gene function studies of the *S. sclerotiorum* secretome, *SsCVNH* was not a unique example to show that the secreted proteins have diverse biological functions during multiple developmental stages of *S. sclerotiorum* (data not shown here). These results suggest a scenario in which different groups of secreted proteins synergistically function during various developmental stages.

Our result showed that SsCVNH was a secreted protein with a CVNH domain. Cyanovirin-N (CVN) has previously been identified only in the aqueous extracts from the cyanobacterium *Nostoc ellipsosporum*^39^. Based on our PSI-BLAST search in the NCBI non-redundant protein sequence database, CVNHs are found in a restricted range of eukaryotic organisms as diverse as the fern *Ceratopteris richardii*, the filamentous ascomycetes (e.g., *Tuber borchii*, *Neurospora*, *Magnaporthe*, *Sclerotinia*, *Botrytis*, *Aspergillus*, *Fusarium*, *Colletotrichum* and *Trichoderma*) and the basidiomycetes (e.g., *Rhizoctonia solani*) but are lacking in other lineages (data not shown). This patchy organism distribution suggests the acquisition of the CVNH domain by some organisms after the separation of the main evolutionary lineages and the occurrence of lateral gene transfer events. CVNH-containing organisms have strikingly different lifestyles, ranging from symbiotic, saprotrophic to pathogenic. Meanwhile, in these organisms, there are both sclerotium-producing fungi and non-sclerotium-producing fungi. These phenomena suggest that the CVNHs are not sufficient for infection and sclerotial development but are necessary in some cases. CVNH-related proteins in ferns and cyanobacteria are found from the secretome. However, owing to data limitations, previous studies reported that no secretion signals were identified in any of the fungal CVNHs in filamentous ascomycetes, and only three types of domain architectures of CVNHs have been reported^31^,^34^. Due to the increasing availability of genomic sequences, we find there are more than three types of domain architectures of CVNHs, including SsCVNH with a signal peptide in the N-terminus before the CVNH domain, thereby indicating that these CVNHs may also be secreted proteins in filamentous ascomycetes. Our result further confirmed this hypothesis and supplemented the knowledge concerning the domain architectures of CVNHs.

## Materials and Methods

### Data collection and bioinformatics analyses

The predicted proteomes of *M. oryzae*, *F. graminearum*, *F. oxysporum, F. verticillioides*, *P. nodorum*, *P. tritici-repentis*, *V. dahliae, B. cinerea*, *S. sclerotiorum*, *U. maydis*, *P. graminis*, *P. triticina*, *P. striiformis*, *S. cerevisiae* and *S. pombe* were downloaded from the Broad Institute (http://www. broadinstitute.org/science/projects/projects). The predicted proteomes of *C. fulvum* and *M. larici-populina* were obtained from the DOE Joint Genome Institute (JGI) site^40^ and the predicted proteome of *B. graminis* was downloaded from BluGen (http://www. blugen.org/)^41^. The microarray data of *M. larici-populina* and *P. graminis* used in this study were downloaded from Gene Expression Omnibus (GEO) at NCBI (http://www.ncbi.nlm.nih.gov/geo/), and their accession numbers are GSE21624 and GSE25020, respectively^27^,^28^. Protein structure modeling was performed using the Phyre2 server^42^ (http://www.sbg.bio.ic.ac.uk/phyre2/html/page.cgi?id=inde7x) and rendered using UCSF Chimera^43^. COBALT^44^ (http://www.ncbi.nlm.nih.gov/tools/ cobalt/cobalt.cgi?link_loc=BlastHomeAd) was used to generate the amino acid alignment, which was viewed and edited with “Jalview” software^45^. The CAZymes were identified by the Hmmscan program in the HMMER 3.0 package^46^ using the family-specific HMM profiles of CAZymes from the dbCAN database^47^. The sub-classification of CAZymes (PCW-degrading enzymes, FCW-degrading enzymes and their respective related CBMs) was performed according to Amselem *et al.*^35^. The SignalP^48^ was used to identify secreted proteins. In this study, we defined cysteine-rich, small, secreted proteins as the secreted proteins consisting of at least 76 aa and that are less than 300 aa in length and contain at least 4% cysteine residues. Gene expression patterns were clustered using “cluster”^49^, and the hierarchical clustering algorithm was used based on the average-linkage method^50^. The dendrogram and colored image were produced by “javaTreeview”^51^. The functional enrichment analyses were performed according to FungiFun2^52^.

### Bacterial and fungal strains, plant and culture conditions

The virulent *S. sclerotiorum* wild-type strain Ep-1PNA367 was used and routinely cultured on potato dextrose agar (PDA, Difco, Detroit, MI, USA) at a neutral pH at 20 °C. *S. sclerotiorum* transformants were cultured on PDA amended with 80 μg/ml hygromycin B (Calbiochem, San Diego, CA, USA). *Escherichia coli* strain DH5α was used to propagate all of the plasmids, and *A. tumefaciens* strain EHA105 was used for the transformation of fungi. Seedlings of *A. thaliana* (ecotype Columbia-0) were grown in the greenhouse at 20 ± 2 °C for one month under a 12 h light/dark cycle with 70% relative humidity.

### Sample preparation, RNA extraction, cDNA production and DGE analysis

For DGE analysis, the Ep-1PNA367 strain was grown or treated under different conditions, and samples were collected for RNA extraction during the following stages: (i) Vegetative stage: fresh mycelia of the Ep-1PNA367 strain were placed on a cellophane membrane overlaid onto PDA medium at 20 °C, and the mycelia were collected at 12, 24, 36, 48 and 60 h; (ii) Sclerotial formation stage: the colonies growing on the cellophane membrane overlaid onto PDA medium were further incubated under the same conditions and then the cultures were collected at 84, 96, 108, 120 and 132 h; (iii) Early stages of infection: fresh hyphal fragments of the Ep-1PNA367 strain were overlaid onto sterilized cheese cloth, which was overlaid onto the leaves of the *A. thaliana* ecotype Columbia-0, followed by inoculation at 20 °C with 100% relative humidity, and then, the cheese cloth with hyphae was rolled up from the leaves at 9 h and 12 h; (iv) Sclerotial myceliogenic germination stage: sclerotia were surface sterilized with sodium hypochlorite and were placed onto a PDA plate at 20 °C to induce myceliogenic germination; the sclerotia were harvested when approximately 50% had germinated; (v) Sclerotial carpogenic germination stage: sclerotia were dried at room temperature and pretreated in a freezer (4–6 °C) for up to one month; then, the sclerotia were surface sterilized and placed onto wet sterilized sands in a plate at 15 °C to induce carpogenic germination; the sclerotia were harvested when approximately 50% germinated (stipes having only emerged from the sclerotia); and (vi) Early apothecial formation stage: sclerotia were allowed to grow in the same incubator, and the stipes were cut and collected immediately before apothecium formation for RNA extraction. Finally, different time-point samples from the vegetative stage, sclerotial formation stage and infection stage were pooled together in equal quantities for RNA extraction. For qRT-PCR analysis, the samples used for validation of the DGE data were prepared independently under the same conditions described above. To evaluate the expression levels of *SsCVNH* in different transformants, the transformants and the wild-type strain were cultured on PDA at 20 °C for 7 dpi before total RNA extraction. This time-point was selected for qRT-PCR analysis because the expression of *SsCVNH* peaks at 7 dpi. For both DGE and qRT-PCR analysis, total RNA extraction was conducted according to Zhu *et al.*^23^. Briefly, total RNA was isolated with TriZOL reagent (Invitrogen, Carlsbad, USA) according to the manufacturer’s protocols. The total RNA was incubated for 30 min at 37 °C with 10 units of DNase I (Roche Applied Science, Shanghai, China) to remove residual genomic DNA. The first cDNA strand was synthesized from 1.0 μg of total RNA by Moloney murine leukemia virus reverse transcriptase (Promega, Madison, USA) using oligo(dT)_18_ primers. For DGE analysis, tag-based transcriptome sequencing methods were used to perform the DGE (Solexa/Illumina, Shenzhen, China) analysis. The DGE raw sequences were transformed into clean tags and subsequently mapped to the *S. sclerotiorum* transcript database from the Broad Institute (http://www.broadinstitute.org/annotation/genome /sclerotinia_sclerotiorum/MultiDownloads.html). All of the clean tags were mapped to the reference sequences with no more than 1 nt mismatch allowed. Clean tags mapped to reference sequences from multiple genes were filtered. The remaining clean tags were designated as unambiguous clean tags. The number of unambiguous clean tags for each gene was calculated prior to normalization to the number of transcripts per million clean tags (TPM)^53^. The complete expression datasets are available at the GEO database (NCBI) as series GSE65301.

### Identification of differentially expressed genes

For DGE experiment, rigorous algorithms were developed to identify differentially expressed genes (or significantly regulated genes) between the two cDNA libraries according to Audic *et al.*^54^. In this study, the P-value and false discovery rate (FDR) were manipulated to determine differentially expressed genes^55^. An FDR ≤ 0.001 and an absolute value of log_2_Ratio ≥ 1 (more than 2.0 fold change) were used as thresholds to determine significant differences in gene expression. For microarray experiment, a Student t test with FDR multiple testing correction was applied to the data using the INVEX web^56^. Transcripts with an adjusted P value < 0.05 and an absolute value of log_2_Ratio ≥ 1 (more than 2.0 fold change) in transcript level were considered as significantly differentially expressed.

### Quantification of gene expression by qRT-PCR

QRT-PCR was used to validate the DGE data and evaluate the expression levels of *SsCVNH* in different transformants. The mRNA transcripts were measured using a SYBR Green I real-time RT-PCR assay in a CFX96™ real-time PCR detection systems (Applied Biosystems). The thermal cycling conditions were 95 °C for 2 min for predegeneration, 40 cycles of 95 °C for 10 s for denaturation, 58 °C for 20 s for annealing and 72 °C for 20 s for extension. All of the reactions were run in triplicate by monitoring the dissociation curve to control the dimers. The *S. sclerotiorum* β-tubulin gene was used as a normalizer^25^. After amplification, data acquisition and analysis were performed using the Bio-Rad CFX Manager^TM^ Software (version 2.0). The 2^−ΔΔ*CT*^ method was chosen as the calculation method^57^. The difference in the cycle threshold (CT) values of the genes and the housekeeping gene β-tubulin (called ΔC_T_) was calculated as follows: ΔΔCT = (ΔC_T_ of genes at each time point)-(ΔC_T_ of the initial control). See supplementary Table S6 for the primers for qRT-PCR.

### Western blot analysis and the subcellular localization of SsCVNH

To generate the *SsCVNH*-FLAG fusion constructs (Figure S9a), the promoter P_EF−1α_ was PCR amplified using the primers P_EF−1α_ − 1 F/R and subsequently digested with *Xho* I and *Sac* I. The PCR products of *SsCVNH* were amplified with the primers FLAG-SsCVNH F/R and subsequently digested with *Sac* I and *Sma* I. These two fragments were sequentially ligated into the pCH vector through intermediate constructs. The primers are shown in Supplementary Table S6. The SsCVNH-FLAG construct was transformed into the wild-type strain using the *Agrobacterium*-mediated transformation method as previously described^58^. Briefly, the *A. tumefaciens* cells with the SsCVNH-FLAG constructs were diluted in minimal medium^59^ amended with 50 μg/ml kanamycin and incubated overnight at 28 °C. Then the *A. tumefaciens* cells were diluted in induction medium^59^ and incubated for 6 h at 28 °C with gentle shaking, before they were co-cultivated with fresh *S. sclerotiorum* mycelial plugs on a cellophane membrane placed on co-induction medium (induction medium with agar) at 20 °C for 2 days. The cellophane membrane was then transferred to the selective medium [PDA amended with 80 μg/ml hygromycin B and 200 μg/ml cefotaxime sodium (DingGuo, Beijing, China)] after the mycelial plugs were removed. Colonies that were regenerated through the selective medium after incubation at 20 °C for 3–6 days were transferred to the selective medium for subcultivation. Total proteins were extracted from the transformants by cell lysis buffer for western blotting and IP (Beyotime, Wuhan, Hubei, China). A total of 5 μl of ANTI-FLAG M2 monoclonal antibody (Sigma, St Louis, MO, USA) was added to 1 ml of the protein extracts and incubated at room temperature for 2 hours. Next, protein A + G agarose (Beyotime, Wuhan, Hubei, China) was added to the protein extracts and incubated at room temperature for 1 hour prior to collection by centrifugation. The extracts were washed five times with cell lysis buffer, and then protein loading buffer was added for the following western blot analysis. Tissues from onion epidermal cells inoculated with SsCVNH-FLAG-engineered strains were sampled at 36 hpi. The lower epidermis was peeled and cut into 1-cm pieces near the inoculation site and fixed with 4% paraformaldehyde for 10 min. Subsequently, the sample was washed 3 times with 1 × phosphate-buffered saline (PBS, pH 7.4) (5 min each wash), incubated in 0.2% Triton X-100 for 10 min and washed 3 additional times with 1 × PBS (5 min each wash). After removing the Triton X-100, the sections were blocked for 30 min in 1% (w/v) BSA and washed 3 times with 1 × PBS (5 min each wash). The sections were subsequently incubated with primary anti-FLAG-tag mouse monoclonal antibody for 2 h at 37 °C, rinsed 3 times with 1 × PBS (5 min each rinse) and incubated in secondary rhodamine red-X-conjugated (RRX) or fluorescein isothiocyanate (FITC)-conjugated goat anti-mouse IgG for 2 h at 37 °C. Subsequently, the onion tissues from the inoculated lower epidermis were rinsed, viewed and photographed using a confocal laser scanning microscope (OLYMPUS® microscope FV1000). The 570 nm and 492 nm absorption laser lines with corresponding appropriate specific emission filter sets were used when imaging RRX and FITC, respectively.

### Construction of RNAi vectors and the transformation of *S. sclerotiorum*

Two strategies described by Nguyen^60^ and Yu^58^ were adopted to construct the *S. sclerotiorum* RNAi vectors. Briefly, a 336 bp fragment from *SsCVNH* was amplified with the primers RNAi-SsCVNH F/R from the *S. sclerotiorum* cDNA library and (i) directly ligated into the pCXDPH vector digested by *Xcm* I (New England Biolabs, Beverly, MA, USA) to produce the pRNAi-1 vector (Figure S9b); and (ii) digested with two pairs of suitable enzymes, followed by ligation of both of the digested fragments into the pCIT vector in the inverse orientation. Finally, the fusion fragment of PtrpC-*SsCVNH*-intron-*SsCVNH*-TtrpC was digested with *Sac І* and *Xho І* and subsequently ligated into the pCH vector^58^ to produce the pRNAi-2 vector (Figure S9c). The primers used to generate these RNAi vectors are shown in Supplementary Table S6. The *Agrobacterium*-mediated transformation method used to transform *S. sclerotiorum* is described above.

### Characterization of the *SsCVNH*-silenced transformants

To evaluate virulence, at least nine individual, fully expanded leaves of *Brassica napus* and tomato were detached and inoculated with a single 0.5-cm diameter fresh mycelium-colonized PDA plug of each silenced transformant and the wild-type strain for moisture culture at 20 °C. Disease severity was measured using the average lesion diameter at 48 hpi. To assay growth rates, the virulent wild-type strain and the silenced transformants were cultivated on PDA at 20 °C for 3 days. Mycelial agar discs were collected from the active colony edge and inoculated in the center of the PDA Petri dish at 20 °C prior to examination of hyphal growth. After growth on PDA at 20 °C for 48 h, the tip hyphal morphology of the virulent wild-type strain and the silenced transformants was observed under a light microscope. Each experiment was performed at least three times.

## Additional Information

**How to cite this article**: Lyu, X. *et al.* Comparative genomic and transcriptional analyses of the carbohydrate-active enzymes and secretomes of phytopathogenic fungi reveal their significant roles during infection and development. *Sci. Rep.* **5**, 15565; doi: 10.1038/srep15565 (2015).

## Supplementary Material

Supplementary Table S1

Supplementary Table S2

Supplementary Table S3

Supplementary Table S4

Supplementary Table S5

Supplementary Table S6

Supplementary Figure S1-9
