# Supplementary material for: Comparative genomic and transcriptional analyses of the carbohydrate-active enzymes and secretomes of phytopathogenic fungi reveal their significant roles during infection and development
Source: Sci Rep. 2015 Nov 4;5:15565. doi: 10.1038/srep15565 (PMC4632110; doi:10.1038/srep15565)
Supplement: Supplementary Figure S1-9 [file srep15565-s7.pdf]

Comparative genomic and transcriptional analyses of the carbohydrate-active enzymes and secretomes of phytopathogenic fungi reveal their significant roles during infection and development

Xueliang Lyu[1,2], Cuicui Shen[1,2], Yanping Fu[2], Jiatao Xie[2], Daohong Jiang[1,2], Guoqing Li[1,2], Jiasen Cheng[1,2*]

1 State Key Laboratory of Agricultural Microbiology, Huazhong Agricultural University, Wuhan 430070, Hubei Province, China

2 The Provincial Key Lab of Plant Pathology of Hubei Province, College of Plant Science and Technology, Huazhong Agricultural University, Wuhan 430070, Hubei Province, China

* Corresponding author:

Jiasen Cheng, Tel: +86-27-87280487, E-mail: jiasencheng@mail.hzau.edu.cn

Supplementary materials

Figure S1. Sequencing quality evaluation of the six cDNA libraries of *S. sclerotiorum*. The six libraries represent transcripts from the stages of sclerotial development (a), vegetative growth (b), sclerotial myceliogenic germination (c), sclerotial carpogenic germination (d), apothecium formation (e) and infection (f), respectively.

Figure S2. Sequencing saturation analysis of the six cDNA libraries. Statistics of the genes mapped by all the clean tags and unambiguous clean tags from the stages of sclerotial development (a), vegetative growth (b), myceliogenic germination (c), carpogenic germination (d), apothecium formation (e) and infection (f), respectively.

Figure S3. The identification of differentially expressed genes in the DGE data. Scatter plots of differentially expressed genes during sclerotial development (a), infection (b), myceliogenic germination (c), carpogenic germination (d) and apothecium formation (e), respectively, compared with the vegetative growth stage. The red and green dots indicate the significantly up-regulated and down-regulated genes, respectively, under the threshold of $|\log_2 Ratio| \geqslant 1$ and FDR $\leqslant 0.001$. (f). The detailed numbers of differentially expressed genes during sclerotial development, infection, myceliogenic germination, carpogenic germination and apothecium formation.

Figure S4. QRT-PCR validation of the DGE data. The TPM values in the DGE data and the relative expression values from qRT-PCR results were compared. Relative quantitation was performed to measure the changes in target gene expression

in the six cDNA libraries. The quantity of target gene cDNA measured by qRT-PCR was normalized to β-tubulin cDNA within each reaction. The relative abundance of target gene cDNA from the vegetative growth stage was arbitrarily assigned a value of 1.0. The standard error of the qRT-PCR is shown. All of these genes were randomly selected. Δ and * indicate that these genes are secreted protein-encoding genes and CAZyme-encoding genes, respectively.

Figure S5. Comparative analysis of secreted proteins in biotrophic fungi, hemibiotrophic fungi and necrotrophic fungi. The numbers of secreted proteins (SP), small secreted proteins (SSP) and cysteine-rich, small secreted proteins (CR-SSP) were plotted.

Figure S6. Expression cluster analysis of secreted protein-encoding genes. The TPM values were used for the expression cluster analysis. Red, green and grey indicate high expression, low expression and no expression, respectively. Top, stage tree; left, gene tree. Expression values are indicated in log2 scale.

Figure S7. The functional enrichment analysis of significantly up-regulated secreted protein-encoding genes of *S. sclerotiorum*. The figure shows all of the enriched FunCat functional categories during different developmental stages. The whole genome of *S. sclerotiorum* was used as the background. An adjusted p-value (Benjamini-Hochberg procedure) of < 0.05 in the modified Fisher's exact test was selected as the significance criteria.

Figure S8. *SsCVNH*-silenced transformants showing significantly reduced virulence on detached tomato leaves. Virulence was evaluated according to the

lesion diameter at 20 °C for 48 h. Three independent replications were performed. The values are presented as the means ± s.d. Different letters in the graph indicate statistical significance, P = 0.05.

Figure S9. Graphical representation of the constructs used in this study. (a). Graphical representation of the *SsCVNH*-FLAG fusion constructs used for immunolocalization. The *SsCVNH*-FLAG fusion was expressed under the control of the $P_{EF\text{-}1\alpha}$ promoter and the *trpC* terminator. SP indicates the signal peptide of SsCVNH. (b). Construction of the pRNAi-1 vector targeted against *SsCVNH*. Partial *SsCVNH* fragment was amplified using the corresponding primers from the *S. sclerotiorum* cDNA library and then inserted between the *N. crassa trpC* promoter P*trpC* and the *A. nidulans gpd* promoter P*gpd*. P*trpC* and P*gpd* are in a reverse orientation in this vector. (c). Construction of pRNAi-2 vector targeted against *SsCVNH*. The fused *SsCVNH*-intron-*SsCVNH* fragment was inserted between the *A. nidulans trpC* promoter P*trpC* and terminator T*trpC*. The two fragments of *SsCVNH* are the same, but they are in a reverse orientation in this vector. The intron is from *G. zeae*.

Table S1. The detailed numbers of PCW- and FCW-degrading CAZymes and related CBMs in each class in the 16 phytopathogenic fungi and two yeasts.

Table S2. The expression profiles of the genes encoding CAZymes (a) and secretome (b) of *S. sclerotiorum*. TPM, normalized clean tag number. The genes encoding CAZymes and secreted proteins not present in this table are the genes whose expression was not detected in the DGE.

Table S3. The identified differentially expressed genes encoding PCW- and FCW-degrading CAZymes and respective related CBMs during different developmental stages of *S. sclerotiorum*. Red: up-regulated genes; green: down-regulated genes. Fold changes are shown in parentheses.

Table S4. The identified differentially expressed genes encoding PCW- and FCW-degradation-associated CAZymes and respective related CBMs during the germination and infection of *M. larici-populina* and *P. graminis* urediniospores. (a). The differentially expressed genes were identified during the germling stage and different infection stages of *M. larici-populina* urediniospores on poplar leaves, compared with the dried-urediniospores in vitro. (b). The differentially expressed genes were identified during the germling stage and the infection of *P. graminis* urediniospores on barley and wheat, respectively, compared with the urediniospores in vitro. Fold changes are shown in parentheses.

Table S5. The GO functional enrichment analyses of the secretome of *S. sclerotiorum*. For each developmental stage, secreted protein-encoding genes that are significantly up-regulated, compared with the vegetative growth stage, were used for the GO functional enrichment analysis. The whole genome of *S. sclerotiorum* was used as the background. An adjusted p-value (Benjamini-Hochberg procedure) of < 0.05 in the modified Fisher's exact test was selected as the significance criteria.

Table S6. Primers used in this study.

Figure S1

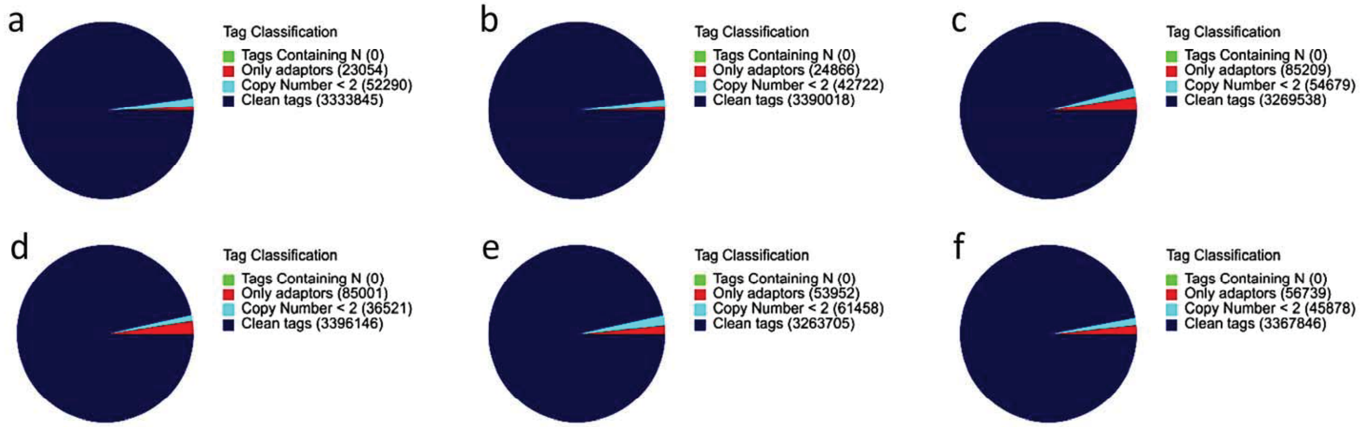
a
Tag Classification
Tags Containing N (0)
Only adaptors (23054)
Copy Number < 2 (52290)
Clean tags (3333845)
b
Tag Classification
Tags Containing N (0)
Only adaptors (24866)
Copy Number < 2 (42722)
Clean tags (3390018)
c
Tag Classification
Tags Containing N (0)
Only adaptors (85209)
Copy Number < 2 (54679)
Clean tags (3269538)
d
Tag Classification
Tags Containing N (0)
Only adaptors (85001)
Copy Number < 2 (36521)
Clean tags (3396146)
e
Tag Classification
Tags Containing N (0)
Only adaptors (53952)
Copy Number < 2 (61458)
Clean tags (3263705)
f
Tag Classification
Tags Containing N (0)
Only adaptors (56739)
Copy Number < 2 (45878)
Clean tags (3367846)

Figure S2

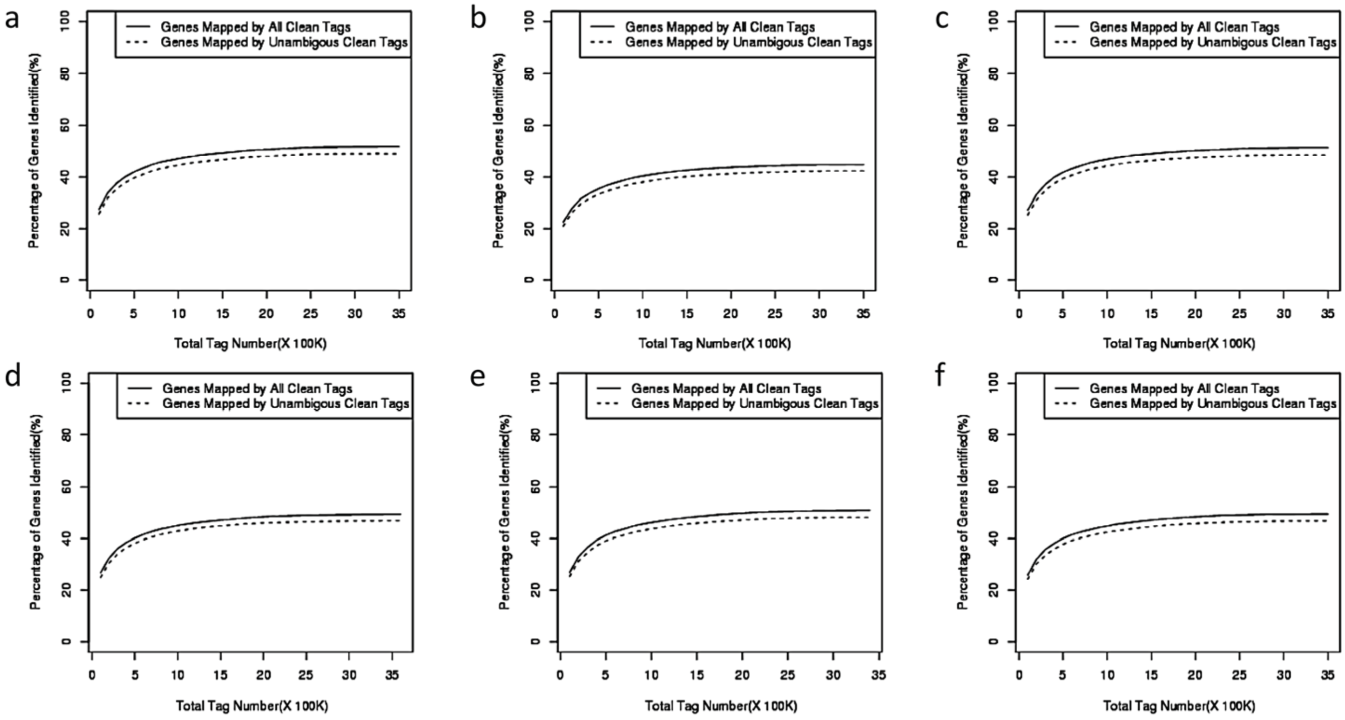
a
b
c
d
e
f
Genes Mapped by All Clean Tags
Genes Mapped by Unambigous Clean Tags
Percentage of Genes Identified(%)
Total Tag Number(X 100K)

Figure S3

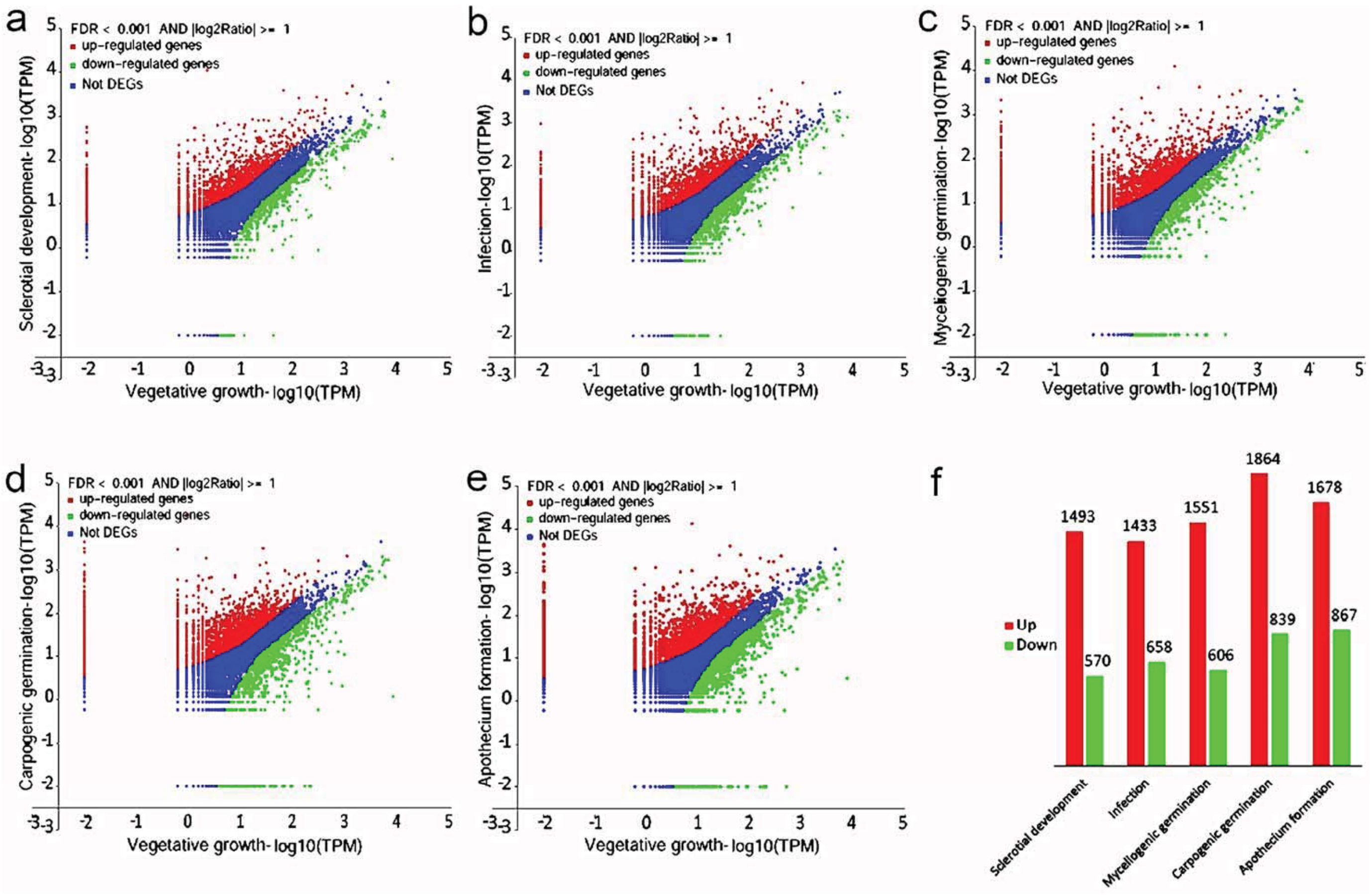
a
FDR < 0.001 AND |log2Ratio| >= 1
up-regulated genes
down-regulated genes
Not DEGs
Sclerotial development- log10(TPM)
Vegetative growth- log10(TPM)
b
FDR < 0.001 AND |log2Ratio| >= 1
up-regulated genes
down-regulated genes
Not DEGs
Infection-log10(TPM)
Vegetative growth- log10(TPM)
c
FDR < 0.001 AND |log2Ratio| >= 1
up-regulated genes
down-regulated genes
Not DEGs
Mycellogenic germination- log10(TPM)
Vegetative growth- log10(TPM)
d
FDR < 0.001 AND |log2Ratio| >= 1
up-regulated genes
down-regulated genes
Not DEGs
Carpogenic germination-log10(TPM)
Vegetative growth- log10(TPM)
e
FDR < 0.001 AND |log2Ratio| >= 1
up-regulated genes
down-regulated genes
Not DEGs
Apothecium formation- log10(TPM)
Vegetative growth- log10(TPM)
f
Up
Down
1493
570
1433
658
1551
606
1864
839
1678
867
Sclerotial development
Infection
Mycellogenic germination
Carpogenic germination
Apothecium formation

Figure S4

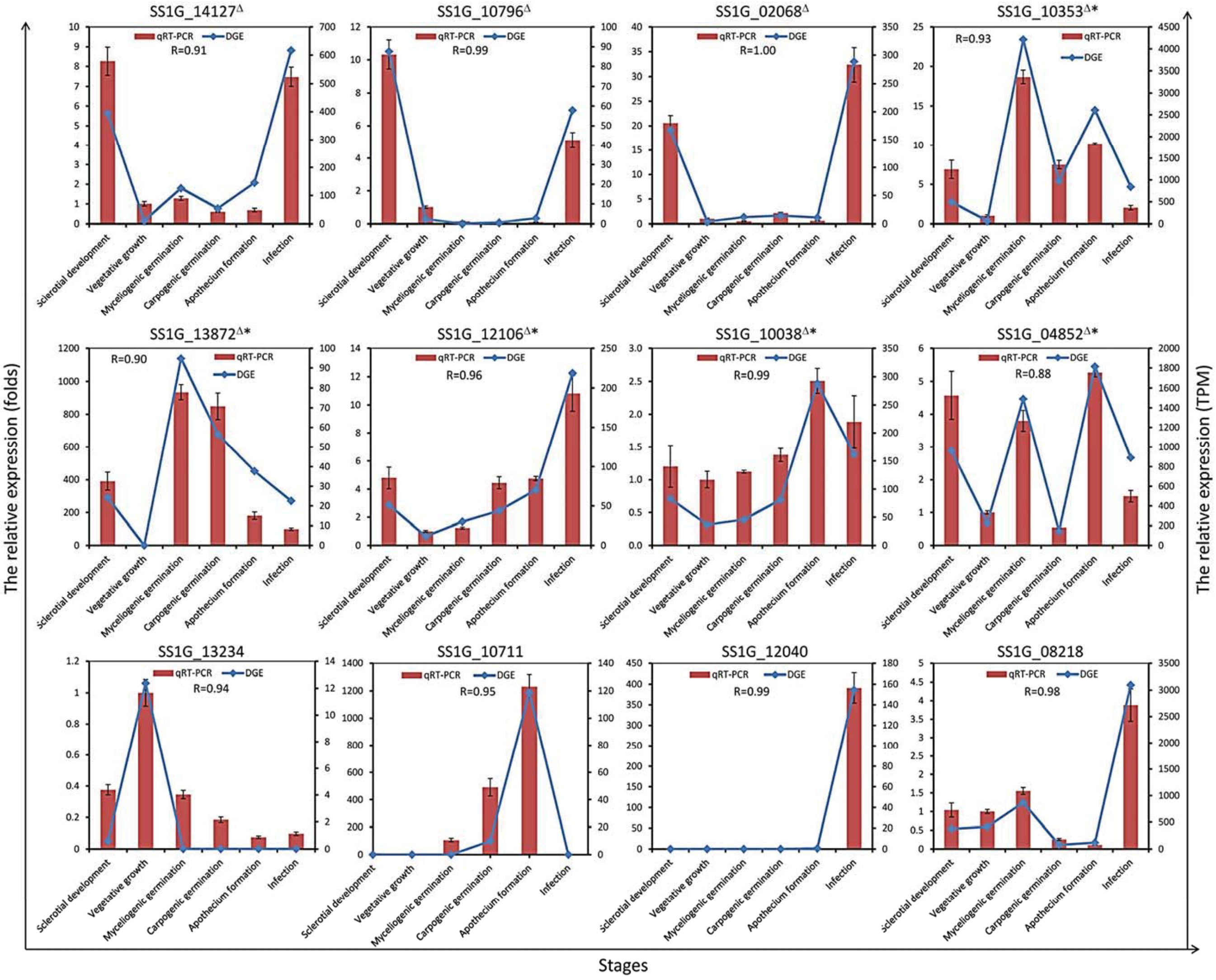
SS1G_14127$^{\Delta}$
R=0.91
SS1G_10796$^{\Delta}$
R=0.99
SS1G_02068$^{\Delta}$
R=1.00
SS1G_10353$^{\Delta}$*
R=0.93
SS1G_13872$^{\Delta}$*
R=0.90
SS1G_12106$^{\Delta}$*
R=0.96
SS1G_10038$^{\Delta}$*
R=0.99
SS1G_04852$^{\Delta}$*
R=0.88
SS1G_13234
R=0.94
SS1G_10711
R=0.95
SS1G_12040
R=0.99
SS1G_08218
R=0.98
qRT-PCR
DGE
Sclerotial development
Vegetative growth
Myceliogenic germination
Carpogenic germination
Apothecium formation
Infection
The relative expression (folds)
The relative expression (TPM)
Stages

Figure S5

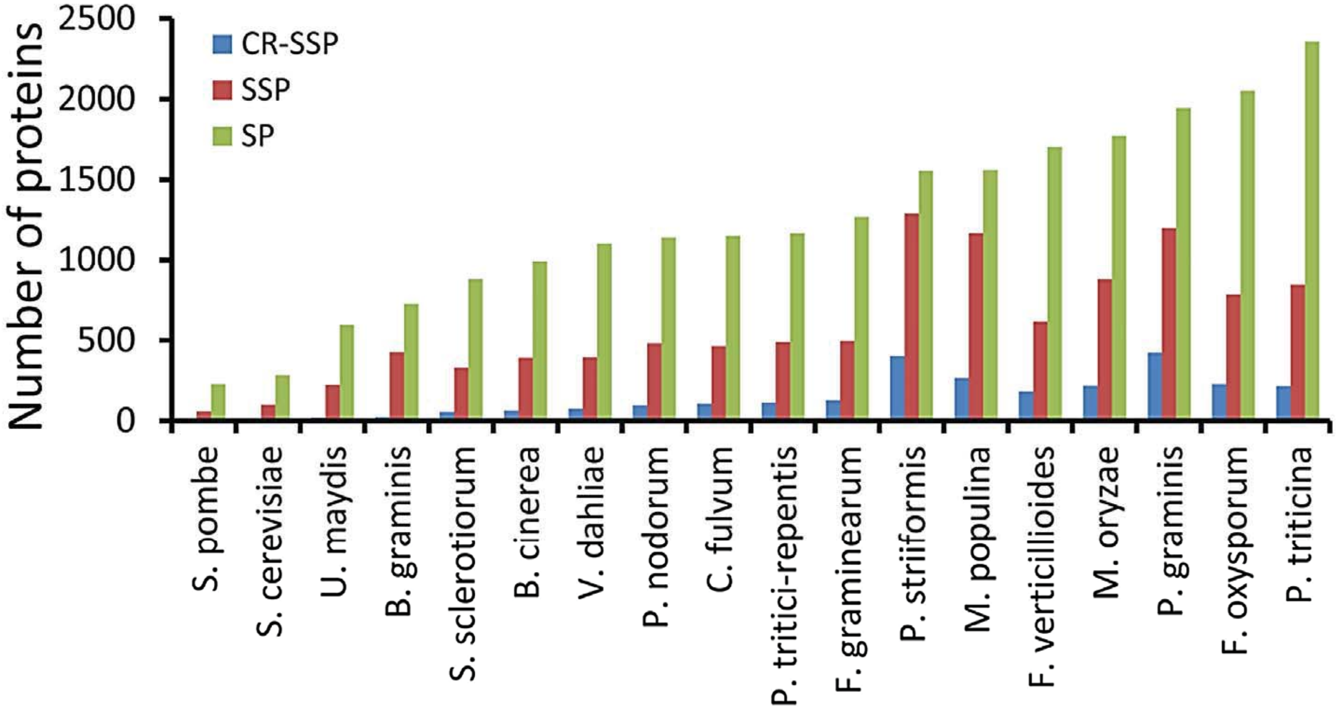
Number of proteins
2500
2000
1500
1000
500
0
CR-SSP
SSP
SP
S. pombe
S. cerevisiae
U. maydis
B. graminis
S. sclerotiorum
B. cinerea
V. dahliae
P. nodorum
C. fulvum
P. tritici-repentis
F. graminearum
P. striiformis
M. populina
F. verticillioides
M. oryzae
P. graminis
F. oxysporum
P. triticina

Figure S6

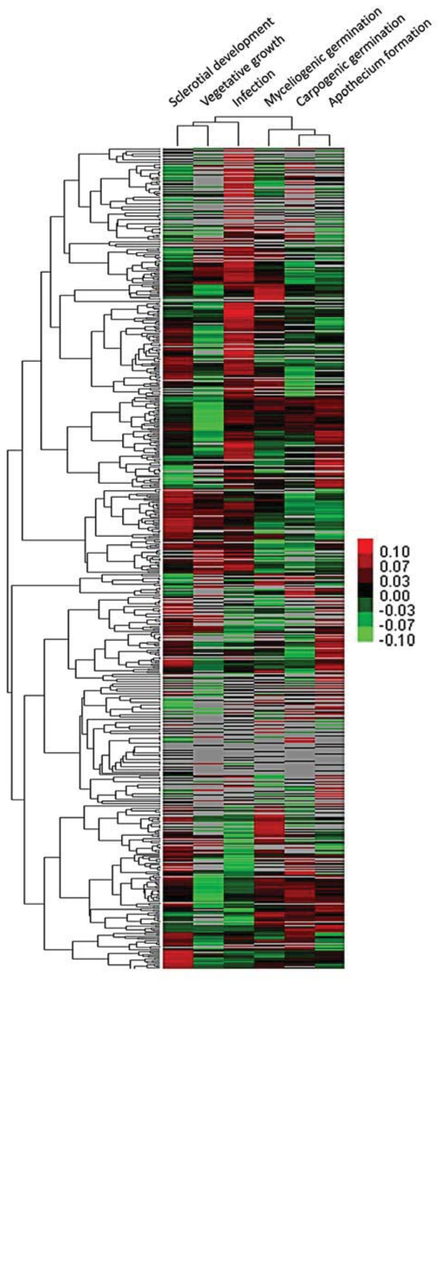

Sclerotial development
Vegetative growth
Infection
Myceliogenic germination
Carpogenic germination
Apothecium formation
0.10
0.07
0.03
0.00
-0.03
-0.07
-0.10

Figure S7

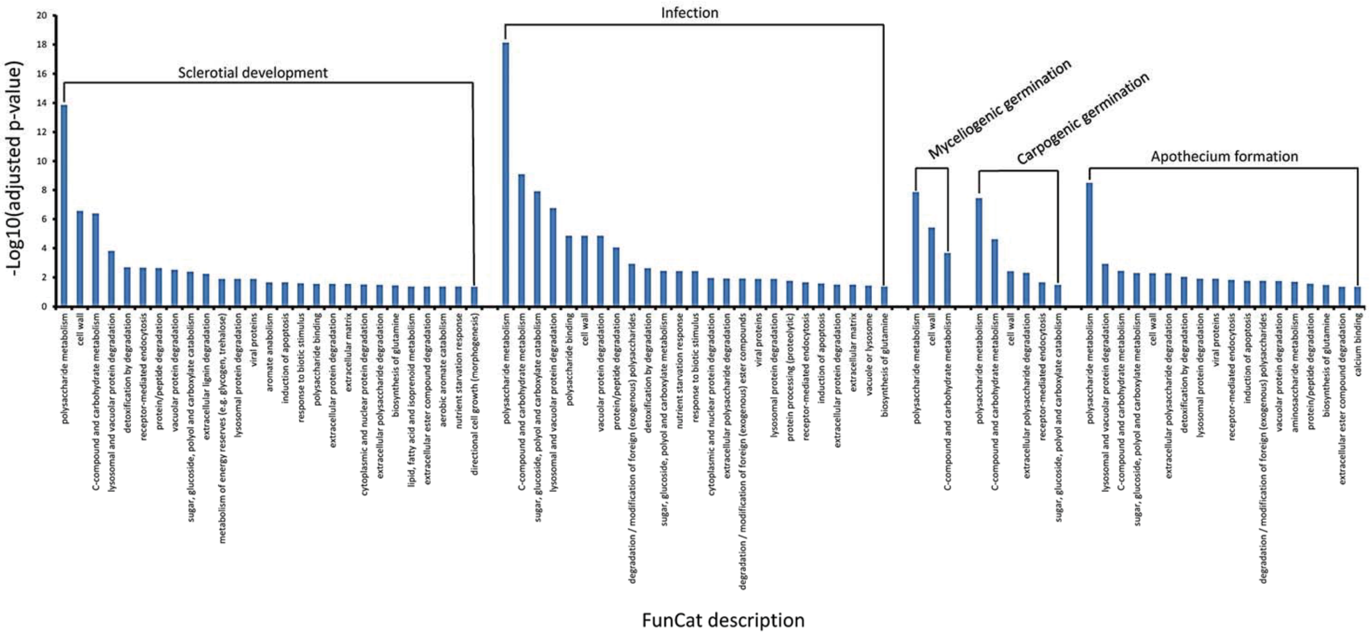

-Log10(adjusted p-value)
0
2
4
6
8
10
12
14
16
18
20
Sclerotial development
Infection
Myceliogenic germination
Carpogenic germination
Apothecium formation
polysaccharide metabolism
cell wall
C-compound and carbohydrate metabolism
lysosomal and vacuolar protein degradation
detoxification by degradation
receptor-mediated endocytosis
protein/peptide degradation
vacuolar protein degradation
sugar, glucoside, polyol and carboxylate catabolism
extracellular lignin degradation
metabolism of energy reserves (e.g. glycogen, trehalose)
lysosomal protein degradation
viral proteins
aromate anabolism
induction of apoptosis
response to biotic stimulus
polysaccharide binding
extracellular protein degradation
extracellular matrix
cytoplasmic and nuclear protein degradation
extracellular polysaccharide degradation
biosynthesis of glutamine
lipid, fatty acid and isoprenoid metabolism
extracellular ester compound degradation
aerobic aromate catabolism
nutrient starvation response
directional cell growth (morphogenesis)
polysaccharide metabolism
C-compound and carbohydrate metabolism
sugar, glucoside, polyol and carboxylate catabolism
lysosomal and vacuolar protein degradation
polysaccharide binding
cell wall
vacuolar protein degradation
protein/peptide degradation
degradation / modification of foreign (exogenous) polysaccharides
detoxification by degradation
sugar, glucoside, polyol and carboxylate metabolism
nutrient starvation response
response to biotic stimulus
cytoplasmic and nuclear protein degradation
extracellular polysaccharide degradation
degradation / modification of foreign (exogenous) ester compounds
viral proteins
lysosomal protein degradation
protein processing (proteolytic)
receptor-mediated endocytosis
induction of apoptosis
extracellular protein degradation
extracellular matrix
vacuole or lysosome
biosynthesis of glutamine
polysaccharide metabolism
cell wall
C-compound and carbohydrate metabolism
polysaccharide metabolism
C-compound and carbohydrate metabolism
cell wall
extracellular polysaccharide degradation
receptor-mediated endocytosis
sugar, glucoside, polyol and carboxylate catabolism
polysaccharide metabolism
lysosomal and vacuolar protein degradation
C-compound and carbohydrate metabolism
sugar, glucoside, polyol and carboxylate metabolism
cell wall
extracellular polysaccharide degradation
detoxification by degradation
lysosomal protein degradation
viral proteins
receptor-mediated endocytosis
induction of apoptosis
degradation / modification of foreign (exogenous) polysaccharides
vacuolar protein degradation
aminosaccharide metabolism
protein/peptide degradation
biosynthesis of glutamine
extracellular ester compound degradation
calcium binding
FunCat description

Figure S8

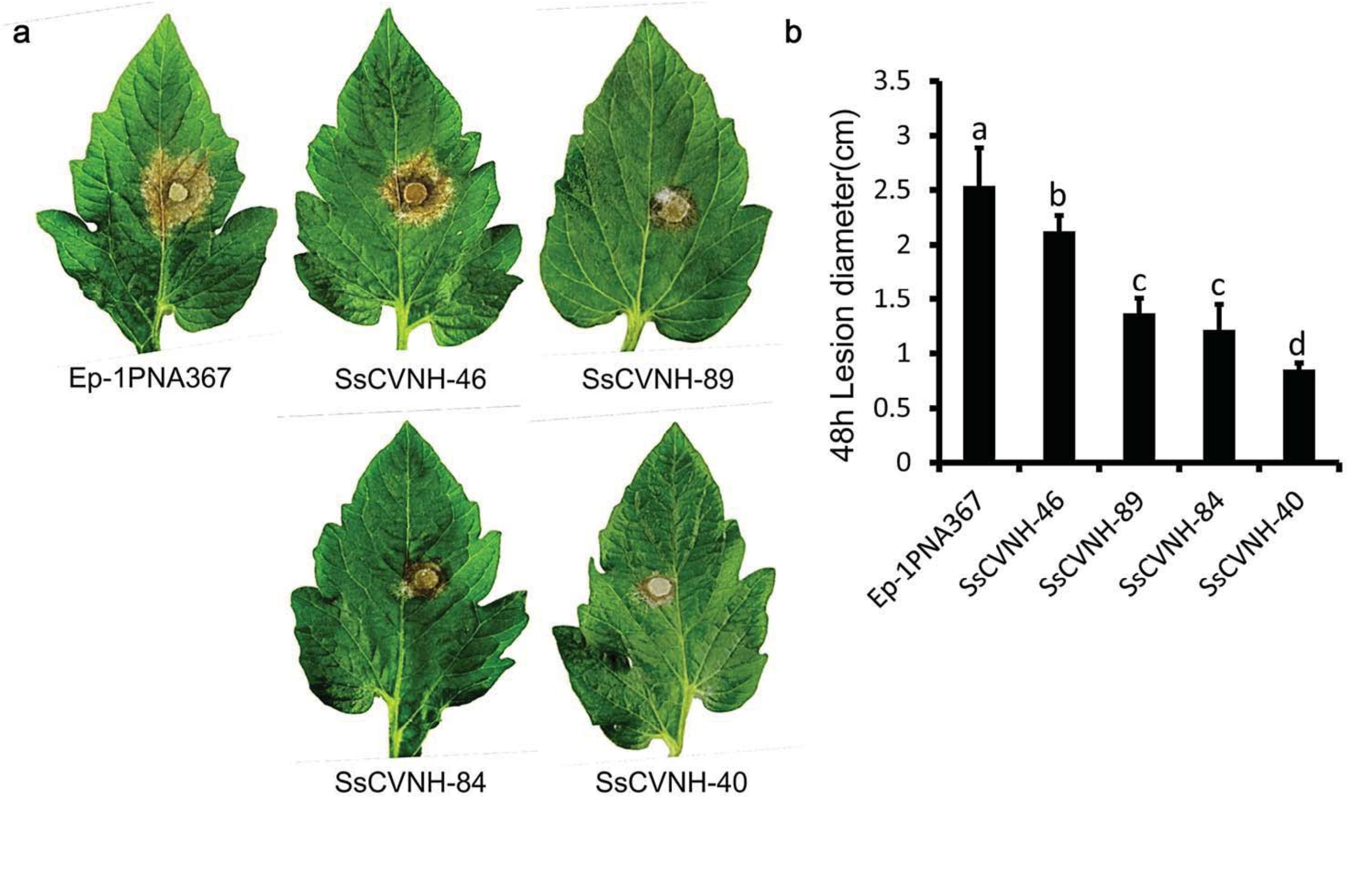
a
Ep-1PNA367
SsCVNH-46
SsCVNH-89
SsCVNH-84
SsCVNH-40
b
48h Lesion diameter(cm)
3.5
3
2.5
2
1.5
1
0.5
0
a
b
c
c
d
Ep-1PNA367
SsCVNH-46
SsCVNH-89
SsCVNH-84
SsCVNH-40

Figure S9

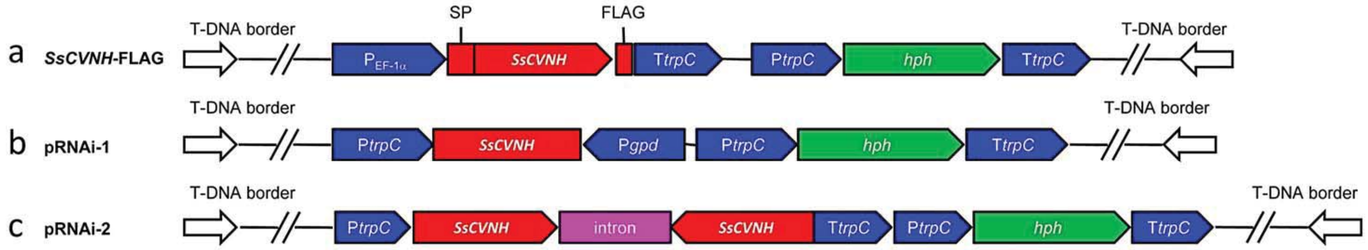
a
SsCVNH-FLAG
T-DNA border
SP
FLAG
T-DNA border
P EF-1α
SsCVNH
TtrpC
PtrpC
hph
TtrpC
b
pRNAi-1
T-DNA border
T-DNA border
PtrpC
SsCVNH
Pgpd
PtrpC
hph
TtrpC
c
pRNAi-2
T-DNA border
T-DNA border
PtrpC
SsCVNH
intron
SsCVNH
TtrpC
PtrpC
hph
TtrpC
